# Saccharomyces cerevisiae Atf1p is an alcohol acetyltransferase and a thioesterase *in vitro*

**DOI:** 10.1002/yea.3229

**Published:** 2017-03-06

**Authors:** Bethany Nancolas, Ian D. Bull, Richard Stenner, Virginie Dufour, Paul Curnow

**Affiliations:** ^1^ School of BiochemistryUniversity of BristolBristolUK; ^2^ School of ChemistryUniversity of BristolBristolUK; ^3^ Bristol Centre for Functional NanomaterialsUniversity of BristolBristolUK; ^4^ BrisSynBioLife Sciences Building, Tyndall AvenueBristolUK

**Keywords:** enzymes, metabolism, protein purification

## Abstract

The alcohol‐*O*‐acyltransferases are bisubstrate enzymes that catalyse the transfer of acyl chains from an acyl‐coenzyme A (CoA) donor to an acceptor alcohol. In the industrial yeast Saccharomyces cerevisiae this reaction produces acyl esters that are an important influence on the flavour of fermented beverages and foods. There is also a growing interest in using acyltransferases to produce bulk quantities of acyl esters in engineered microbial cell factories. However, the structure and function of the alcohol‐*O*‐acyltransferases remain only partly understood. Here, we recombinantly express, purify and characterize Atf1p, the major alcohol acetyltransferase from S. cerevisiae. We find that Atf1p is promiscuous with regard to the alcohol cosubstrate but that the acyltransfer activity is specific for acetyl‐CoA. Additionally, we find that Atf1p is an efficient thioesterase *in vitro* with specificity towards medium‐chain‐length acyl‐CoAs. Unexpectedly, we also find that mutating the supposed catalytic histidine (H191) within the conserved HXXXDG active site motif only moderately reduces the thioesterase activity of Atf1p. Our results imply a role for Atf1p in CoA homeostasis and suggest that engineering Atf1p to reduce the thioesterase activity could improve product yields of acetate esters from cellular factories. © 2017 The Authors. *Yeast* published by John Wiley & Sons, Ltd.

## Introduction

Volatile esters are secondary metabolites produced by yeast and fungi during fermentation (Saerens *et al.,* 2010; Mason and Dufour, 2000) and in plants during fruit ripening (El Hadi *et al.,* 2013). These compounds are important and desirable components of the flavour of fruits and of fermented beverages such as beer and wine (Saerens *et al.,* 2010; Robinson *et al.,* 2014). Understanding the biochemistry of the volatile esters is thus of considerable importance in industrial agriculture, winemaking and brewing. In addition, the enzymes responsible for ester synthesis are targets for the metabolic engineering of cellular ‘factories’ intended to produce fragrances, industrial solvents, fine chemicals, pharmaceuticals and renewable biofuels (Rodriguez *et al.,* 2014; Tashiro *et al.,* 2015; Layton and Trinh, 2014; Tai *et al.,* 2015). However, in many cases the molecular structures and biological functions of these enzymes remain cryptic.

The Saccharomyces cerevisiae alcohol‐*O*‐acetyltransferases (AATs; EC 2.3.1.84) Atf1p and Atf2p are thought to synthesize a range of acetate esters from acetyl‐coenzyme A (CoA) and various alcohols (Saerens *et al.,* 2010; Mason and Dufour, 2000; Verstrepen *et al.,* 2003a). The most important acetate esters from the perspective of fermented foods and beverages are probably ethyl acetate (fruity, solvent aroma), isobutyl acetate (sweet, fruit), isoamyl acetate (banana) and 2‐phenylethyl acetate (rose). Homologues of these two enzymes are also found in other yeasts (Van Laere *et al.,* 2008). From the perspective of yeast biology, AATs are thought to play an important role in detoxifying ethanol and other metabolites, in recycling CoA from acyl‐CoA and in fatty acid metabolism (Saerens *et al.,* 2010). A series of investigations have now defined the contribution of AATs to ester production in S. cerevisiae at the cellular level. Knockout strains of Atf1p and Atf2p exhibit reduced synthesis of numerous acetate esters relative to wild‐type strains and, conversely, overexpression of Atf1p and Atf2p leads to the increased production of acetate esters (Verstrepen *et al.,* 2003b; Fujii *et al.,* 1996; Lilly *et al.,* 2000; Lilly *et al.,* 2006). It appears that, of these two proteins, Atf1p plays the major role in acetate ester synthesis (Verstrepen *et al.,* 2003b); there is evidence that Atf2p is important for sterol detoxification (Tiwari *et al.,* 2007). Although these previous reports provide compelling evidence for the importance of AATs in yeast metabolism, they reveal little about the detailed structure and function of Atf1p and Atf2p. Such an understanding could be provided by studies of purified proteins *in vitro*.

Atf1p is a peripheral membrane protein that is localized to the endoplasmic reticulum and lipid particles via amphipathic domains at the N‐ and C‐termini (Lin and Wheeldon, 2014; Verstrepen *et al.,* 2004). This membrane localization presents a challenge to protein purification because Atf1p must first be extracted from the lipid milieu. Earlier studies found that endogenous Atf1p could be partially isolated by serial column chromatography after washing S. cerevisiae membranes with detergents (Malcorps and Dufour, 1992; Minetoki *et al.,* 1993; Yoshioka and Hashimoto, 1981; Akita *et al.,* 1990). Recombinant Atf1p and Atf2p both form aggregates with significantly diminished activity when overexpressed in Escherichia coli, and this is also the case for at least some plant AATs (Zhu *et al.,* 2015). Here, we adopt an alternative approach in which recombinant Atf1p, including a polyhistidine purification tag, is overexpressed from an autonomous plasmid in a laboratory yeast strain. We show that this recombinant Atf1p can be successfully purified from sedimenting membranes and characterize the activity and substrate specificity of the purified protein.

## Materials and methods

### Materials

Molecular biology reagents were from New England Biolabs. The yeast shuttle vector pYES2/CT and the antibody anti‐V5‐HRP (catalogue number R961–25) were from Life Technologies. Precast acrylamide gels were from NuSep. Acyl‐CoAs, reagents for assays of esterase and thioesterase activity and Thesit were from Sigma‐Aldrich. *n*‐Dodecyl‐β‐d‐maltopyranoside (DDM) was from Anatrace. HisTrap Ni‐NTA columns, PD‐10 desalting columns and Superdex 200 10/300 GL size exclusion column were from GE Healthcare. Spin concentrators were from Sartorius. LumiGLO chemiluminescent reagents were from Cell Signaling Technology.

### Gene cloning

A synthetic gene corresponding to S. cerevisiae *atf1* (open reading frame YOR377w) was purchased from Eurofins Genomics. The stop codon was not synthesized and, to allow subcloning, the synthetic gene sequence was flanked by upstream and downstream sites for restriction by BamHI and XbaI, respectively. An internal BamHI site was also removed from the synthetic gene by changing the codon for G476 from GGA to GGT. Gene *atf2* (YGR177c) was amplified by PCR from S. cerevisiae genomic DNA prepared by ethanol precipitation (Knight *et al.,* 2014) using the forward and reverse primers 5′‐atgcggatccatggaagatatagaaggatacgaaccacatatcac‐3′ and 5′‐agtctctagaaagcgacgcaaattcgccgatgg‐3′, respectively. This removes the stop codon and introduces BamHI and XbaI restriction sites for cloning as for a*tf1*. After restriction digestion with BamHI and XbaI, *atf1* and *atf2* were cloned into the multiple cloning site of the yeast expression vector pYES2/CT by cohesive end ligation. This strategy places the cDNA in‐frame with vector sequences that introduce a V5 epitope (GKPIPNPLLGLDST), His_10_ tag (introduced by ourselves to replace the manufacturers His_6_ tag) and stop codon in that order at the carboxyl terminal of the translated protein. For protein expression, plasmids were transformed into the protease‐deficient auxotroph S. cerevisiae strain FGY217 (*MATa*, *ura3–52*, *lys2*Δ*201*, *pep4*Δ) as described (Kota *et al.,* 2007; Gietz and Schiestl, 2007). Site‐directed mutations were made via plasmid PCR with overlapping primers (‘Quikchange’). For mutant H191A the forward and reverse primers were 5′‐agaaaagtggaaaaaatttatctttgtatctaatgcttgcatgtctgatggtcg‐3′ and 5′‐cgaccatcagacatgcaagcattagatacaaagataaattttttccacttttct‐3′, respectively. For D195N the forward and reverse primers were 5′‐ggatcgaagaccgaccattagacatgcaatgattaga‐3′ and 5′‐tctaatcattgcatgtctaatggtcggtcttcgatcc‐3′, respectively. Mutations were confirmed by sequencing.

### Protein expression and purification

Recombinant yeast strains were grown at 30°C with shaking at 250 rpm in selective media without uracil supplemented with glucose. The overexpression of recombinant Atf1p and Atf2p was induced by diluting the cultures to *A*_600_ = 0.4 in selective media containing 2% galactose/0.1% glucose and growing for a further 24 h. Recombinant proteins were ultimately purified from isolated yeast membranes by immobilized metal affinity chromatography. This was carried out essentially as described previously (Knight *et al.,* 2014), but with the purification resin in column format rather than batch mode. Briefly, following cell lysis with a continuous‐flow cell disruptor at 35 kPSI (Constant Systems Ltd, UK), membranes were sedimented by ultracentrifugation at 170000 ***g*** for 1 h. To liberate the overexpressed recombinant proteins, the sedimenting membranes were treated with either 2% DDM or 2% Thesit (hydroxypolyethoxydodecane; also known as C12E9), both of which were previously used to extract endogenous Atf1p (Malcorps and Dufour, 1992). A second ultracentrifugation step was used to remove insoluble material. Detergent‐solubilized proteins were then loaded onto a 1 mL Ni‐NTA HisTrap column equilibrated in Column Buffer (50 mm sodium phosphate, 150 mm NaCl, 5% glycerol, 0.02% Thesit or 0.05% DDM, pH 7.4) plus 20 mm imidazole at a flow rate of 1 mL min^−1^. The resin was washed with 40 vols of Column Buffer plus 60 mm imidazole. Purified protein was eluted in 2.5 vols of Column Buffer plus 0.5 m imidazole at a flow rate of 0.2 mL min^−1^. Imidazole was removed immediately using a PD‐10 desalting column before the protein was concentrated to 2 mg mL^−1^ in a centrifugal concentrator with molecular weight cut‐off of either 30 kDa (Thesit) or 100 kDa (DDM). These ‐purified proteins were confirmed as full‐length recombinant Atf1p by mass spectrometry and used directly without any further purification. Protein concentrations in cell fractions were determined using a detergent‐compatible Lowry assay (BioRad DC Protein Assay) and the concentration of purified recombinant protein was additionally determined by absorbance at 280 nm using a calculated extinction coefficient of 83 770 m ^−1^ cm^−1^ and theoretical molecular weight of 64.8 kDa for the tagged Atf1p (http://web.expasy.org/protparam/).

### Protein analysis

Size exclusion chromatography (SEC), SDS‐PAGE and circular dichroism (CD) were all carried out as described previously (Knight *et al.,* 2014), with detergents at 2–4 × critical micelle concentration (CMC) in all working buffers unless otherwise stated. The SEC column was calibrated against the following protein standards: ovalbumin (molecular weight 44 kDa, Stokes radius 3.1 nm), conalbumin (75 kDa, 3.6 nm), aldolase (158 kDa, 4.8 nm) and ferritin (440 kDa, 6.1 nm). To access lower wavelengths in CD, Atf1p was diluted to 0.2 mg mL^−1^ to give buffer conditions of 10 mm sodium phosphate, pH 7.4, 135 mm NaF, 15 mm NaCl and 5% glycerol. Protein concentration was determined from the absorbance at 280 nm as described above. A 1 mm pathlength cell was freshly cleaned with 2% Hellmanex and washed extensively before loading with 200 µL of Atf1p. Scan parameters on a Jasco J‐1500 instrument were 25°C, 100 nm min^−1^, 1 nm bandwidth, 2 s integration, eight accumulations. The DICHROWEB suite for CD analysis was accessed at http://dichroweb.cryst.bbk.ac.uk.

### Biochemical acyltransferase assay

Acyltransferase activity was determined using a coupled assay as described previously (Dunn *et al.,* 2013; Knight *et al.,* 2014), except that the *α*‐ketoglutarate dehydrogenase complex was purified by D. Denton (McCormack and Denton, 1979) and was used at 0.01 U per reaction. Atf1p was used at 0.5 µm in 400 µL reaction volume at 25°C . The initial (linear) rates of enzyme progress curves under different substrate conditions were determined by linear regression. These rates were fit to a hyperbolic Michaelis–Menten curve by non‐linear regression with GraphPad Prism. Thin‐layer chromatography was performed in a mobile phase of 9:1:0.1 dichloromethane–methanol–formic acid (*v*/v) after spotting 1 µL of each reaction on a silica gel F_254_ plate (Merck Millipore). The plate was developed with iodine.

### Thioesterase assay

Purified recombinant Atf1p was incubated at 25°C with 25 µm *p*‐nitrophenylthioacetate or *p‐*nitrophenylacetate in 50 mm sodium phosphate buffer, pH 7.4, 150 mm NaCl. The concentration of *p*‐nitrophenol generated by thioesterase activity was determined by absorbance at 400 nm relative to a standard curve.

### Gas chromatography–mass spectrometry

Volatile products of the enzymatic acyltransferase reaction were analysed by solid phase microextraction (SPME) gas chromatography–mass spectrometry (GC–MS). The extraction procedure, instrument parameters and data analysis were as described previously (Plutowska and Wardencki, 2008; Knight *et al.,* 2014) except that the MS scanning was carried out in the range *m*/*z* 30–550. Typical reaction mixtures were 0.2 mm acyl‐CoA, 2.5% alcohol (at least 400‐fold molar excess) and 2–4 µm Atf1p in 50 mm sodium phosphate buffer, pH 7.4. For the most volatile esters (ethyl acetate, ethyl butyrate and isobutyl acetate) a lower‐temperature programme was used in which samples were heated from 50 to 70°C at 5°C min^−1^ before being ultimately heated to 240°C for injection, with MS scanning in the range *m*/*z* 5–300. In all cases the molecular ions are minor peaks or are not observed, and products are instead identified based upon characteristic fragmentation patterns (Christie, 2014; Knight *et al.,* 2014; Sharkey *et al.,* 1959).

## Results and discussion

### Protein expression and purification

Atf1p and Atf2p were cloned into a commercial expression vector (pYES2/CT) to enable the expression of these recombinant proteins after galactose induction. Each of these constructs was designed to include two additional components at the protein carboxyl terminus. The first of these was an artificial V5 epitope, to allow the detection of overexpressed protein by western blotting. The second component was a 10‐residue polyhistidine tag (His_10_) to allow protein purification by immobilized metal affinity chromatography. For convenience we refer here to the recombinant Atfp–V5–His_10_ constructs simply as Atf1p or Atf2p. The full sequence of each of these expression constructs is shown in Figure S1 in the Supporting Information.

**Figure 1:**
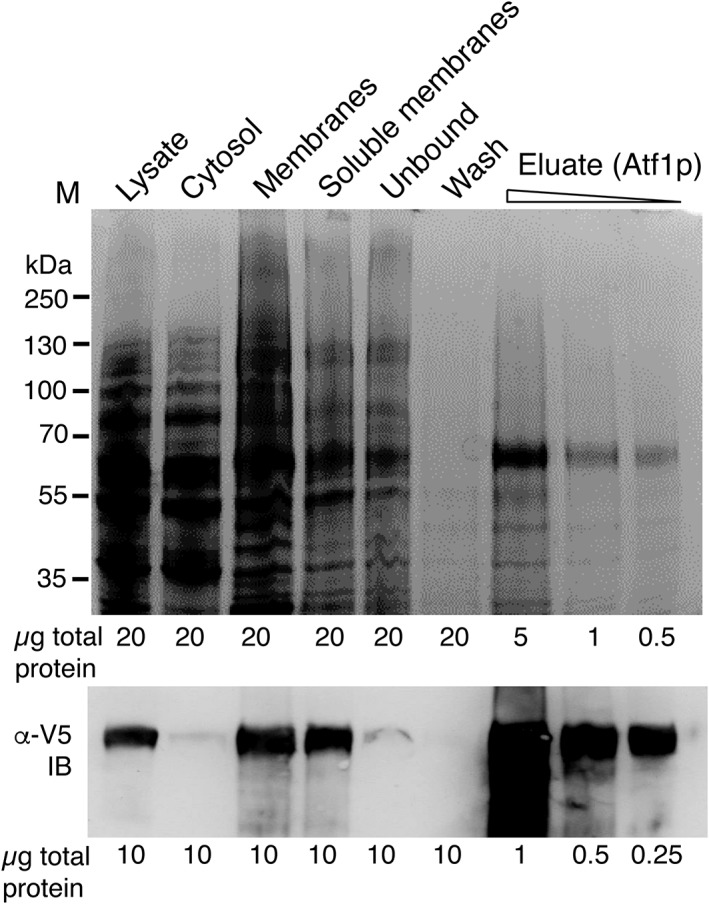
Expression of Atf1p and purification after solubilization with the detergent Thesit. Top panel, SDS‐PAGE gel of cell fractions and column purification fractions as shown, stained with Coomassie Brilliant Blue. Bottom panel, western blot (IB) of the same samples with anti‐V5‐HRP (*α*‐V5) confirming both the membrane localization of recombinant Atf1p and the success of detergent solubilization and purification. The protein loadings used for Coomassie staining and western blotting are shown

We found that the recombinant Atf1p could be successfully expressed by S. cerevisiae (Figure 1). In contrast, recombinant Atf2p was not overexpressed for unknown reasons. Atf2p is already known to be overexpressed with difficulty in other recombinant systems (Zhu *et al.,* 2015). Given the apparent challenges associated with Atf2p, we thus focus here on characterizing Atf1p. We used ultracentrifugation to fractionate the recombinant cell and used western blotting to show that, as expected, the expressed Atf1p was found to localize to the sedimenting membrane fraction. We treated these membranes with either of the detergents Thesit or DDM to form soluble protein–detergent–lipid complexes. The solublilized membranes were applied to an Ni^2+^ affinity resin and recombinant Atf1p was purified to homogeneity in a single step (Figure 1). Western blotting was used to demonstrate successful solubilization and column binding, with soluble Atf1p (Figure 1, *Soluble membranes*) being depleted from the column flow‐through (Figure 1, *Unbound*) and retained during column washing. After elution, the purified recombinant Atf1p migrated as a single band on an SDS‐PAGE gel at an apparent molecular weight close to the calculated theoretical weight of 65 kDa. Some minor bands were observed at lower molecular weight. These were also detected by western blotting and so presumably represent minor species of Atf1p with unusual migration, perhaps caused by anomalous SDS binding. The major band at 65 kDa was excised and confirmed to be recombinant Atf1p by mass spectrometry (Figure S2 in the Supporting Information). Typical yields from this purification were 1 mg of Atf1 per litre of recombinant yeast culture.

### Characterization of Atf1p

**Figure 2:**
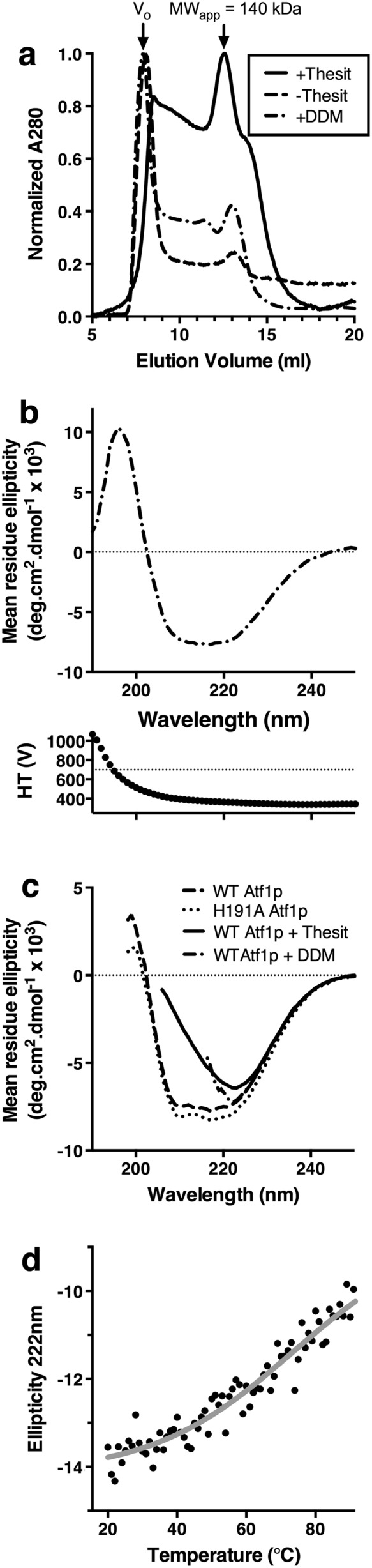
Characterization of purified Atf1p. (a) Size‐exclusion chromatography shows a tendency for Atf1p to aggregate with a significant peak in the void volume (Vo) corresponding to an apparent molecular weight of >600 kDa. This is somewhat alleviated by the detergent Thesit. (b) CD of Atf1p in low detergent NaF buffer, with the spectrum characteristic of mixed *α*/*β* structure. The high‐tension voltage (HT) signal is shown underneath, with the quality threshold of 700 V indicated by a horizontal line. (c) Qualitative CD of Atf1p in purification buffers, and comparison with mutant H191A. Secondary structure is apparently lost at detergent concentrations above the critical micelle concentration. Data are truncated at the lowest wavelength for which HT <700 V. (c) Thermal stability of wild‐type Atf1p determined by CD melt

The oligomeric state of Atf1p was characterized by Size exclusion chromatography (SEC) and the secondary structure and thermal stability by circular dichroism (CD). The results are shown in Figure 2. SEC chromatograms were generally heterogenous, suggesting non‐specific protein aggregation (Figure 2a). This aggregation was somewhat reduced by maintaining Thesit within the SEC buffer, which resulted in a relatively smaller peak in the void volume and a relatively larger peak at an elution volume corresponding to an apparent molecular weight of 140 kDa (Stokes radius of 4.6 nm). This peak is consistent with a protein–detergent complex comprising an Atf1p monomer of 65 kDa associated with the Thesit micelle, which is approximately 83 kDa (Strop and Brunger, 2005). DDM made only a modest difference to the chromatogram vs. no detergent, suggesting that this detergent does not prevent protein aggregation.

The secondary structure of Atf1p was predicted from the primary sequence using the PSIPRED web server (Jones, 1999; Buchan *et al.,* 2013). The results, shown in Figure S3 in the Supporting Information, predict that Atf1p will adopt a mixed α/β structure. This is similar to the secondary structure content of other *O*‐acetyltransferases (Leslie *et al.,* 1988; Cai *et al.,* 2004; Govindasamy *et al.,* 2004), including the BAHD superfamily to which plant AATs belong (Ma *et al.,* 2005; Walker *et al.,* 2013; Unno *et al.,* 2007). It is informative to directly compare the secondary structure prediction of Atf1p with a similar analysis of an AAT from papaya, VpAAT1, because in the latter case the PSIPRED prediction can be benchmarked against the secondary structure observed in a homology model (Protein Model Database ID: PM0079098; Morales‐Quintana *et al.,* 2011)). For VpAAT1 the homology model and the structure prediction are in reasonably good agreement. Comparing VpAAT1 and Atf1p shows that the number, order and sequence position of predicted secondary structure elements are broadly similar. The major difference between the two proteins is the presence of an additional amphipathic helix predicted at the *N*‐terminal of Atf1p. This helix is known to play a role in localizing Atf1p to the ER and lipid droplet (Lin and Wheeldon, 2014) and so may be a novel feature of Atf1p compared with the plant AATs.

The secondary structure of Atf1p was determined experimentally by CD in two independent experiments. In the first of these, data for wild‐type Atf1 were recorded in NaF buffer to obtain higher‐quality CD spectra that extend to lower wavelengths (Kelly *et al.,* 2005). In the second set of experiments, data were recorded in the same buffer as used for functional assays, which was not optimized for CD measurements. Nonetheless, these experiments allowed for direct qualitative comparison between wild‐type Atf1p and mutant H191A, as well as comparisons with the same proteins at high detergent concentrations. In all cases a photomultiplier high‐tension voltage (HT) signal of <700 V was taken as a quality threshold (Kelly *et al.,* 2005).

The CD spectrum of wild‐type Atf1p diluted into detergent‐free buffer was as expected for a protein with mixed α/β secondary structure, with major deflections at 222 and 208 nm (Figure 2b). We analysed this data using the tools implemented at the DICHROWEB server (Whitmore and Wallace, 2004, 2008; Lobley *et al.,* 2002). The best results with lowest RMSD (0.095) were obtained with CONTIN‐LL (van Stokkum *et al.,* 1990) using the SMP180 dataset (Abdul‐Gader *et al.,* 2011), although qualitatively similar results were obtained with other analysis methods and datasets. This analysis suggested that the relative proportion of different secondary structure elements in Atf1p was 0.23 α‐helix, 0.25 β‐strand, 0.12 Turn and 0.40 Unordered. Accurately deconvoluting the composite circular dichroic spectra of mixed α/β structures is known to be challenging (Whitmore and Wallace, 2008). However, the experimental results were in reasonable agreement with the secondary structure prediction (Figure S3 in the Supporting Information), which suggested that, of the 558 residues in recombinant Atf1p, 159 (0.28) will be in α‐helix and 76 (0.14) will be in β‐strand.

Figure 2c shows qualitative CD data suggesting that the secondary structure of Atf1p was sensitive to the presence of detergent, with a substantial loss of the 208 nm signal in 0.02% Thesit and 0.05% DDM. This result is consistent with previous suggestions that DDM can inactivate Atf1p (Malcorps and Dufour, 1992). Figure 2(c) also shows that introducing the active‐site mutation H191A (see below) did not perturb the gross protein secondary structure. This is consistent with prior results showing that introducing the same mutant to a homology model did not perturb the structure of VpAAT1 *in silico* (Morales‐Quintana *et al.,* 2013). Recombinant Atf1p was reasonably stable to increases in temperature up to 40°C with a substantial loss in secondary structure thereafter (Figure 2d).

It thus appears that, while including Thesit in working buffers reduces protein aggregation (Figure 2a), detergents at concentrations above the critical micelle concentration (CMC) also influence the secondary structure of Atf1p (Figure 2c). Because of this, in all of the functional studies described below we dilute the purified protein to work at detergent concentrations below the CMC.

### Acetate ester production by Atf1p

**Figure 3:**
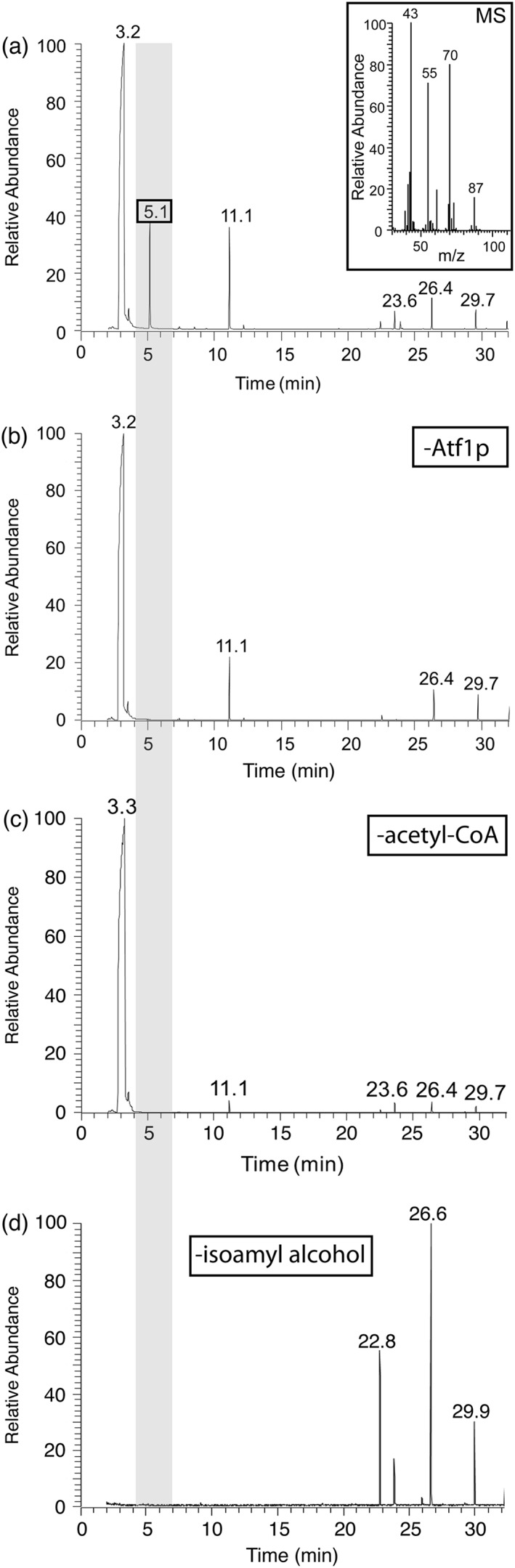
Production of a volatile ester by Atf1p. (a) Incubating purified recombinant Atf1p, isoamyl alcohol and acetyl‐CoA generates a GC peak at 5.1 min (boxed) that is confirmed as isoamyl acetate by coupled MS (inset). As expected, the molecular ion, which would have *m*/*z* = 130, is not observed. Instead, the characteristic fragmentation peaks are: *m*/*z* = 43, CH_3_C=O^+^, acetyl cation; *m*/*z* = 87, C_2_H_4_OC(=O)CH_3_^+^, loss of alkyl free radical from molecular ion; *m*/*z* = 70, [(CH_3_)_2_CHCH=CH_2_]^•+^, elimination of neutral acetic acid from molecular ion; *m*/*z* = 55, CH_3_C(=CH_2_)CH_2_^+^, cleavage of the preceding alkyl radical cation; the minor unlabelled peak at *m*/*z* = 61 is protonated acetic acid from the ‘McLafferty +1’ or ‘2H’ rearrangement (Sharkey *et al.,* 1959; McGoran *et al.,* 1996; McLafferty and Tureček, 1993). (b–d) The isoamyl acetate product is not formed spontaneously in controls lacking one of either enzyme (−Atf1p), acetyl‐CoA (−acetyl‐CoA) or alcohol (−isoamyl alcohol)

To study the alcohol acetyltransferase activity of Atf1p, we systematically screened this enzyme against a panel of alcohol and acyl‐CoA cosubstrates and detected volatile ester products with GC–MS (Figure 3 and S4–S6 in the Supporting Information). We found that Atf1p could use acetyl‐CoA to synthesize acetate esters from all of the alcohols tested, producing ethyl acetate, isoamyl acetate, isobutyl acetate, butyl acetate, hexyl acetate, heptyl acetate and octyl acetate. However, we could not detect any volatile products in reactions in which Atf1p was tested against other acyl‐CoAs (C3, C4, C5, C6, C8, C10, C12). Thus we confirm previous observations that the alcohol acyltransferase activity of Atf1p is promiscuous with regard to alcohol but relatively specific for acetyl‐CoA (Verstrepen et al., 2003a; Fujii *et al.,* 1996; Lilly *et al.,* 2000, Lilly *et al*., 2006; Yoshioka and Hashimoto, 1981; Rodriguez *et al.,* 2014).

### Biochemical assays of Atf1p

**Scheme 1:**
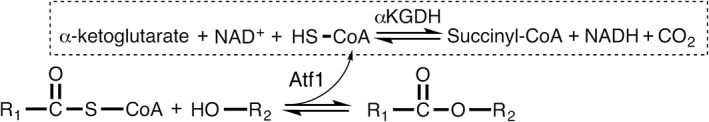
Atf1p alcohol acetyltransferase reaction and the coupled system (boxed) used to study enzyme kinetics. *α*KGDH, *α*‐Ketoglutarate dehydrogenase complex

We next measured the enzymatic activity of recombinant Atf1p using a coupled assay (Dunn *et al.,* 2013). The principle of the assay is that the turnover of acyl‐CoA by Atf1p generates free CoA, which in turn is a substrate for α‐ketoglutarate dehydrogenase complex (αKGDH). The activity of the coupled αKGDH reduces NAD^+^ to NADH and so generates an absorption signal at 340 nm. This assay thus measures CoA release, rather than ethyl ester synthesis. This is shown in Scheme 1.

**Figure 4:**
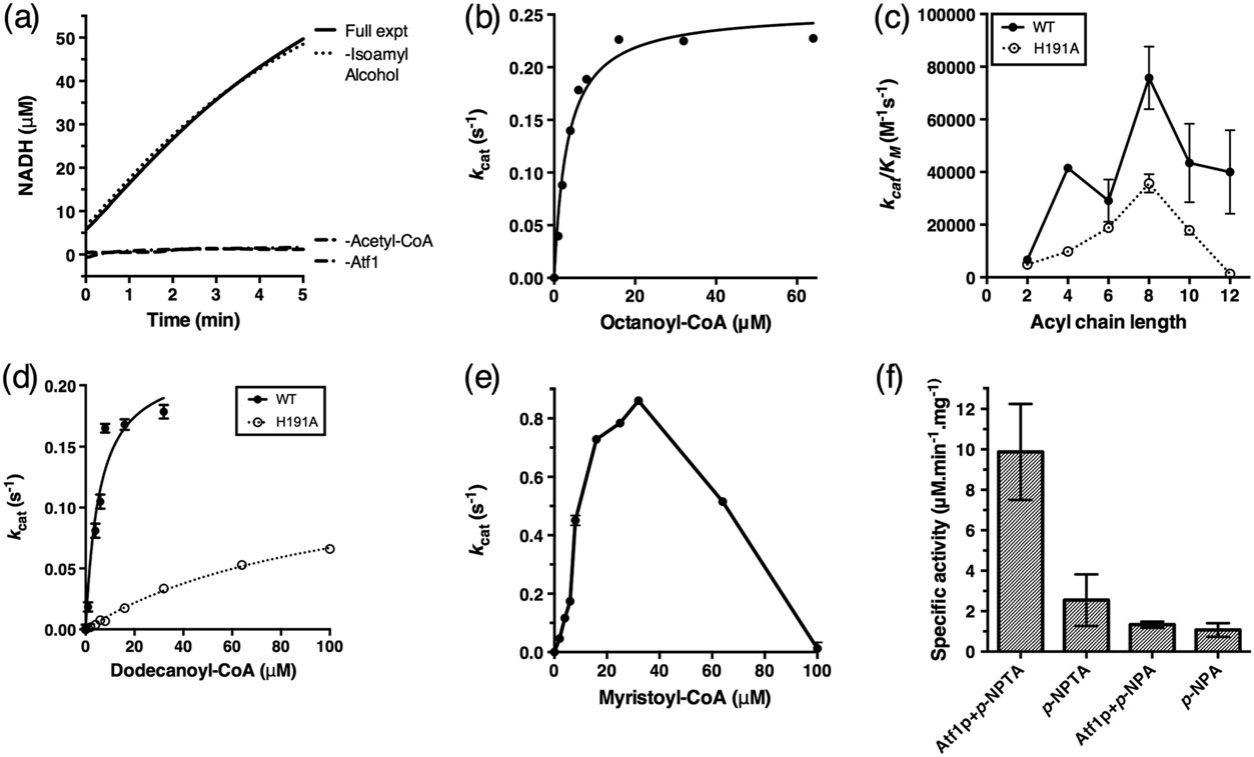
Biochemical assays of Atf1p. (a) The activity of Atf1p against acetyl‐CoA in a coupled assay does not require the alcohol cosubstrate. (b) Example of the initial (linear) rates from Atf1p assays fit to the Michaelis–Menten equation (solid line). (c) Atf1p is active against a range of acyl‐CoAs, with greatest catalytic efficiency toward C8 (octanoyl) substrates. Mutating the conserved active site residue H191 decreases activity overall and (d) significantly influences activity against C12 substrates, where *K*_M_ is increased 20‐fold vs. wild‐type. (e) Apparent product inhibition in wild‐type Atf1p at C14 chain lengths. (f) Atf1p is active as a thioesterase but is not a general esterase. Data are mean ± SD (*n* = 3)

We first recorded the activity of Atf1p in the presence of both acetyl‐CoA and alcohol substrates as well as in controls where one or other of the substrates was omitted. Surprisingly, we found that the activity of Atf1p was virtually identical regardless of the presence or absence of an alcohol cosubstrate (Figure 4a). It thus appears that Atf1p can efficiently hydrolyse acetyl‐CoA and we confirmed the formation of hydrolysis products by thin‐layer chromatography (Figure S7 in the Supporting Information). We then tested the activity of Atf1p against acyl‐CoA substrates of up to C14 chain length and found that, with the exception of C14, enzyme kinetics from these assays could be successfully fit to the Michaelis–Menten equation (Figure 4b). These results generally agree with the conclusions of Lin and colleagues, who recently also reported a broad substrate spectrum for Atf1p in recombinant cell lysates (Lin *et al.,* 2016). Although Atf1p could hydrolyse all of the acyl‐CoA substrates tested, we observed a clear preference (highest *k*_cat_/*K*_M_ ) for C8 chain lengths (Figure 4c). This was largely the result of changes in *K*_M_, which decreased with increasing chain length up to C8 (octanoyl) and then began to increase again at higher chain lengths (Table S1 in the Supporting Information). For example, *K*_M_ was 61 ± 9 μm for acetyl‐CoA, 25 ± 1 μm for butyryl‐CoA, 12 ± 3 μm for hexanoyl‐CoA and 3 ± 1 μm for octanoyl‐CoA. In contrast *k*_cat_ was relatively similar for all of the substrates tested, being in the range 0.2–1 s^−1^ with no obvious trend. These results are similar to those that we previously obtained with the S. cerevisiae alcohol acyltransferase Eht1p. This enzyme synthesized medium‐chain‐length fatty acid ethyl esters but was also an effective acyl‐CoA hydrolase (Knight *et al.,* 2014). The major differences between the enzymes are that Eht1p had a narrower substrate range and an even clearer preference for octanoyl‐CoA. Eht1p was also more efficient, with *k*_cat_/*K*_M_ for Eht1p and Atf1p against octanoyl‐CoA being 15 × 10^4^ and 8 × 10^4^ m ^−1^ s^−1^, respectively.

Atf1p contains the conserved HXXXD(G) active site motif common to CoA‐dependent *O‐*acyltransferases generally and to the plant AATs within the BAHD superfamily in particular (D'Auria, 2006). This is in contrast to Eht1p, which has a classical Ser‐His‐Asp catalytic triad (Saerens *et al.,* 2006; Knight *et al.,* 2014). To test the importance of the HXXXDG motif in Atf1p protein structure and function, we introduced the independent mutations H191A and D195N. The mutation D195N was apparently highly deleterious to Atf1p and this mutant could not be recombinantly expressed. In contrast, mutant H191A was expressed at levels only slightly below those of wild‐type protein and could be purified from cell membranes using the same methods. Values of *k*_cat_/*K*_M_ for H191A were about half that of the wild‐type at most of the acyl chain lengths tested (Figure 4c and Table S1 in the Supporting Information). This was caused by a reduction in *k*_cat_, with *K*_M_ being reasonably similar between the mutant and wild‐type proteins between C2 and C10. However, a marked difference was observed for the C12 substrate. Although *k*_cat_ was half that of wild‐type, *K*_M_ for H191A was increased to 108 ± 18 μm vs. 6 ± 2 μm for wild‐type (Figure 4d). This observed increase in *K*_M_ is a hallmark of competitive inhibition, and probably arises from product inhibition. This effect was even more pronounced for the C14 acyl‐CoA, with both wild‐type Atf1p and H191A showing a rapid decrease in the catalytic rate at higher substrate concentrations leading to no detectable activity (Figure 4e). We note that the high concentrations of substrate required to observe this effect *in vitro* are unlikely to be physiological (Fægerman and Knudsen, 1997).

Our data suggest that, in aqueous buffers *in vitro*, Atf1p is able to efficiently hydrolyse the acyl‐CoA thioester bond. To confirm this thioesterase activity of Atf1p we performed enzyme assays with the generic thioesterase substrate *p*‐nitrophenylthioacetate (*p*‐NPTA). We found that Atf1p was indeed active in cleaving this thioester (Figure 4f), albeit with specific activity reduced ~40‐fold vs. authentic acyl‐CoA substrates. Atf1p was not active against the equivalent hydrolase substrate *p*‐nitrophenylacetate, demonstrating the selectivity of Atf1p towards thioesters.

## Discussion

Atf1p belongs to a large and functionally diverse group of enzymes that act as CoA‐dependent *O*‐acyltransferases. As a subset of this broader grouping, the AATs (EC 2.3.1.84) are found in fungi and plants with the plant enzymes being classified as members of the BAHD superfamily (D'Auria, 2006). Classical examples of the *O*‐acyltransferases include chloramphenicol acetyltransferase (Lewendon *et al.,* 1994), choline acetyltransferase (Cai *et al.,* 2004) and carnitine acetyltransferase (Wu *et al.,* 2003). One major conserved sequence feature across the *O*‐acyltransferases, which otherwise can have rather low sequence homology, is the active site HXXXD(G) motif where H and D are absolutely conserved. This conserved motif is present in the plant and fungal AATs, being HXXXDG in Atf1p. Otherwise, Atf1p lacks the BAHD superfamily signature motif DFGWG that is found in plant AATs (D'Auria, 2006). Despite the disparate range of reactions that are catalysed by the *O*‐acyltransferases, structural studies suggest that these enzymes adopt a common architecture that positions the HXXXD(G) active site residues at the interface of two connected, structurally related, domains. Although within this overall fold there are differences in the number and the arrangement of secondary structure elements, the domains generally feature a central β‐sheet region surrounded by α‐helices. Molecular modeling has strongly suggested that plant AATs share this fold (Morales‐Quintana *et al.,* 2011, 2012, 2013; Navarro‐Retamal *et al.,* 2016; Galaz *et al.,* 2013). Substrate access to the buried active site from either face of the enzyme is provided via two linked solvent channels that extend to the protein surface, and in many cases it appears that each of the tunnels is dedicated to one of the individual cosubstrates (Cai *et al.,* 2004). We anticipate that Atf1p will adopt a similar fold, which is supported by the data presented here (Figure 2 and Figure S3 in the Supporting Information). We note that this overall fold is not expected to be disrupted by active site mutation H191A (Morales‐Quintana *et al.,* 2013) and this is consistent with the experimental data in Figure 2(c).

Modelling and experiments with plant AATs have provided support for a non‐covalent ternary complex mechanism similar to that found in chloramphenicol acetyltransferase (Morales‐Quintana *et al.,* 2013; Navarro‐Retamal *et al.,* 2016; Galaz *et al.,* 2013). This posits that the active‐site histidine acts as a general base by forming a hydrogen bond between the imidazole NE2 and the alcohol hydroxyl. This facilitates nucleophilic attack by the deprotonated hydroxyl at the carbonyl of the acyl‐CoA thioester (Kleanthous and Shaw, 1984; Lewendon *et al.,* 1994). It follows from this model that the active site histidine is expected to be essential for catalysis by the AATs, and indeed this is confirmed by experimental and computational studies of the plant enzymes (Morales‐Quintana *et al.,* 2013; Navarro‐Retamal *et al.,* 2016; Galaz *et al.,* 2013). These models also suggest that the active site aspartic acid is not directly involved in catalysis and does not interact with the histidine in a catalytic dyad, but rather makes other contacts that maintain the structure of the active site (Morales‐Quintana *et al.,* 2013). This important structural role may explain why Atf1p mutant D195N could not be expressed.

In this context the data in Figure 4 are unexpected, since they show that mutating the supposedly essential active site residue H191 in Atf1p has only a modest effect in reducing *k*_cat_ by half (Figure 4c). This suggests that the conserved active‐site histidine is not absolutely required for substrate hydrolysis; we did not assess ester production by H191A, and it may be that the alcohol acyltransferase reaction is abolished in this mutant. The H191A mutation leaves *K*_M_ essentially unchanged, and this was anticipated since results with other acyltransferases suggest that the catalytic histidine can play only a minor role in determining substrate binding affinity (Lewendon *et al.,* 1994; Suzuki *et al.,* 2003; Niu *et al.,* 1990; Hsiao *et al.,* 2006). The exception to this general trend is at C14 chain lengths, where the *K*_M_ of H191A is ~20 times larger than that of wild‐type protein. We speculate that H191A may be more sensitive to the onset of the product inhibition that is observed with longer acyl chains (Figure 4e).

There are a very limited number of examples in which an equivalent mutation showed similar effects to that seen here for H191A in Atf1p. Mutating the catalytic histidine in dihydrolipoamide acyltransferase (E2p) from yeast pyruvate dehydrogenase (Niu *et al.,* 1990) had only a marginal effect on *k*_cat_ and no effect on *K*_M_; this stands in contrast to the clear importance of the catalytic His in other E2p proteins (Hendle *et al.,* 1995). Another example is found in VibH, a non‐ribosomal peptide synthetase condensation domain that uses the HXXXD motif to catalyse amide bond formation through a mechanism analogous to acyltransfer. In this case, mutating the catalytic histidine reduced *k*_cat_ by half but did not influence *K*_M_ for one substrate and slightly increased it for the other (Keating *et al.,* 2002). Clearly, further insight into how H191 contributes to catalysis by Atf1p could be obtained through structural biology and we are now actively pursuing this.

We show here that, although Atf1p can only use acetyl‐CoA as a donor for acyltransfer, this enzyme can effectively hydrolyse various acyl‐CoA substrates *in vitro* (Figure 4). It is well established that acyltransferases can be versatile, particularly with regard to the acceptor substrate (Eudes *et al.,* 2016; Landmann *et al.,* 2011; D'Auria, 2006). Medium‐chain acyl‐CoAs can be substrates for some plant AATs and, as observed here, the *K*_M_ for these substrates decreases as the acyl chain length increases (Balbontín *et al.,* 2010; Morales‐Quintana *et al.,* 2013). The unexpected hydrolase activity of Atf1p may have previously been undetected since it does not produce a volatile product and so is not observed by GC–MS experiments on cell cultures. If this activity is relevant *in vivo*, we speculate that it may help to modulate the levels of medium chain length acyl‐CoAs that are released from the stalled fatty acid synthase complex (FAS) in yeast. The products of the yeast FAS are long‐chain fatty acyl‐CoAs, primarily saturated C16 and C18. These have myriad physiological roles (Fægerman and Knudsen, 1997). In particular, these fatty acyl‐CoAs need to be desaturated to C16:1 and C18:1 for lipid synthesis. During fermentation, the reduced activity of the oxygen‐dependent Δ^9^‐fatty acyl‐CoA desaturase Ole1p causes the accumulation of saturated acyl‐CoAs in the cytosol. These bind to, and inhibit, acetyl‐CoA carboxylase and so prevent the formation of malonyl‐CoA, which is the primary substrate of FAS (Sumper, 1974). In the absence of malonyl‐CoA, FAS is arrested, causing the release of partially elongated medium‐chain‐length fatty acyl‐CoAs to the cytosol (Sumper, 1974). It may be that a specific medium‐chain fatty acyl hydrolase activity, such as observed here for Atf1p, is advantageous in controlling the abundance of these medium‐chain‐length acyl‐CoAs. Such an activity would convert medium‐chain acyl‐CoAs to medium‐chain fatty acids that can be removed from the cell and would regenerate free cytosolic CoA (Mason and Dufour, 2000; Bardi *et al.,* 1999; Saerens *et al.,* 2010).

There have been recent efforts to use Atf1p as a component for synthetic biology applications. For example, Tashiro and colleagues used Atf1p as the final step in a metabolic pathway designed to produce the industrial solvent and food additive isobutyl acetate in E. coli (Tashiro *et al.,* 2015). Rodriguez and colleagues also used recombinant Atf1p to turn E. coli into a whole‐cell biocatalyst that can synthesize a range of industrially relevant volatile esters (Rodriguez *et al.,* 2014)*.* Clearly, acyl‐CoA hydrolysis would reduce ester production in such engineered cells. Our results here suggest that engineering Atf1p to favour alcohol acetyltransferase activity over hydrolysis could be an effective means of increasing ester yields in synthetic cell factories.

### Funding and support

There are no competing financial interests. This study was supported by the European Research Council through Starting Grant no. 282101, under the European Union's Seventh Framework Programme, FP7/2007–2013, to P.C. R.S. is supported by EPSRC doctoral training grant EP/L016648/1. Data necessary to support the conclusions are included in the paper and supplement. V.D. is supported by BrisSynBio, a BBSRC/EPSRC Synthetic Biology Research Centre (BB/L01386X/1). The authors thank the UK Natural Environment Research Council for partial funding of the mass spectrometry facilities at Bristol (Contract no. R8/H10/63; http://www.lmsf.co.uk).

## Supporting information


**Supplementary Figure 1.** DNA sequences of recombinant Atf1p and Atf2p
**Supplementary Figure 2.** Mass spectrometry of purified Atf1p
**Supplementary Figure 3.** PSIPRED prediction of the secondary structure of Atf1p
**Supplementary Figure 4.** GC–MS confirming acetate ester synthesis (lower MW volatiles)
**Supplementary Figure 5.** GC–MS confirming acetate ester synthesis (higher MW volatiles)
**Supplementary Figure 6.** GC–MS shows Atf1p is specific to acetyl‐CoA for ester synthesis
**Supplementary Figure 7.** Thin‐layer chromatography confirms acyl‐CoA hydrolysis by Atf1p
**Supplementary Table 1.** Enzyme kinetics
