# Supplementary material for: Saccharomyces cerevisiae Atf1p is an alcohol acetyltransferase and a thioesterase in vitro
Source: Yeast. 2017 Mar 6;34(6):239–51. doi: 10.1002/yea.3229 (PMC5484351; doi:10.1002/yea.3229)
Supplement: Supplementary file 1 — Supplementary Figure 1. DNA sequences of recombinant Atf1p and Atf2p Supplementary Figure 2. Mass spectrometry of purified Atf1p Supplementary Figure 3. PSIPRED prediction of the secondary structure of Atf1p Supplementary Figure 4. GC–MS confirming acetate ester synthesis (lower MW volatiles) Supplementary Figure 5. GC–MS confirming acetate ester synthesis (higher MW volatiles) Supplementary Figure 6. GC–MS shows Atf1p is specific to acetyl‐CoA for ester synthesis Supplementary Figure 7. Thin‐layer chromatography confirms acyl‐CoA hydrolysis by Atf1p Supplementary Table 1. Enzyme kinetics [file YEA-34-239-s001.docx]

**Supplementary Information**

*Saccharomyces cerevisiae* Atf1p is an alcohol acetyltransferase and a thioesterase *in vitro*

Bethany Nancolas^1^, Ian D. Bull^2^, Richard Stenner^1,3^, Virginie Dufour^1,4^ and Paul Curnow^1,4^*

**Affiliations**: ^1^School of Biochemistry, ^2^School of Chemistry, ^3^Bristol Centre for Functional Nanomaterials, University of Bristol, UK. ^4^BrisSynBio, Life Sciences Building, Tyndall Avenue, Bristol, UK

***Corresponding author**. Paul Curnow, School of Biochemistry, Biomedical Sciences Building, University of Bristol, University Walk, Bristol UK BS8 1TD

Email: p.curnow@bristol.ac.uk

**Contents**

Supplementary Figure 1. DNA sequences of recombinant Atf1p and Atf2p

Supplementary Figure 2. Mass spectrometry of purified Atf1p

Supplementary Figure 3. PSIPRED prediction of the secondary structure of Atf1p

Supplementary Figure 4. GC-MS confirming acetate ester synthesis (lower MW volatiles)

Supplementary Figure 5. GC-MS confirming acetate ester synthesis (higher MW volatiles)

Supplementary Figure 6. GC-MS shows Atf1p is specific to acetyl-CoA for ester synthesis

Supplementary Figure 7. Thin-layer chromatography confirms acyl-CoA hydrolysis by Atf1p

Supplementary Table 1. Enzyme kinetics

Supplementary references

**Supplementary Figure 1**

>Atf1-V5-His_10_

**GGATCC**ATGAATGAAATCGATGAGAAAAATCAGGCCCCCGTGCAACAAGAATGCCTGAAAGAGATGATTCAGAATGGGCATGCTCGGCGTATGGGATCTGTTGAAGATCTGTATGTTGCTCTCAACAGACAAAACTTATATCGAAACTTCTGCACATATGGAGAATTGAGTGATTACTGTACTAGGGATCAGCTCACATTAGCTTTGAGGGAAATCTGCCTGAAAAATCCAACTCTTTTACATATTGTTCTACCAACAAGATGGCCAAATCATGAAAATTATTATCGCAGTTCCGAATACTATTCACGGCCACATCCAGTGCATGATTATATTTCAGTATTACAAGAATTGAAACTGAGTGGTGTGGTTCTCAATGAACAACCTGAGTACAGTGCAGTAATGAAGCAAATATTAGAAGAATTCAAAAATAGTAAGGGTTCCTATACTGCAAAAATTTTTAAACTTACTACCACTTTGACTATTCCTTACTTTGGACCAACAGGACCGAGTTGGCGGCTAATTTGTCTTCCAGAAGAGCACACAGAAAAGTGGAAAAAATTTATCTTTGTATCTAATCATTGCATGTCTGATGGTCGGTCTTCGATCCACTTTTTTCATGATTTAAGAGACGAATTAAATAATATTAAAACTCCACCAAAAAAATTAGATTACATTTTCAAGTACGAGGAGGATTACCAATTATTGAGGAAACTTCCAGAACCGATCGAAAAGGTGATAGACTTTAGACCACCGTACTTGTTTATTCCGAAGTCACTTCTTTCGGGTTTCATCTACAATCATTTGAGATTTTCTTCAAAAGGTGTCTGTATGAGAATGGATGATGTGGAAAAAACCGATGATGTTGTCACCGAGATCATCAATATTTCACCAACAGAATTTCAAGCGATTAAAGCAAATATTAAATCAAATATCCAAGGTAAGTGTACTATCACTCCGTTTTTACATGTTTGTTGGTTTGTATCTCTTCATAAATGGGGTAAATTTTTCAAACCATTGAACTTCGAATGGCTTACGGATATTTTTATCCCCGCAGATTGCCGCTCACAACTACCAGATGATGATGAAATGAGACAGATGTACAGATATGGCGCTAACGTTGGATTTATTGACTTCACCCCCTGGATAAGCGAATTTGACATGAATGATAACAAAGAAAATTTTTGGCCACTTATTGAGCACTACCATGAAGTAATTTCGGAAGCTTTAAGAAATAAAAAGCATCTCCATGGCTTAGGGTTCAATATACAAGGCTTCGTTCAAAAATATGTGAACATTGACAAGGTAATGTGCGATCGTGCCATCGGGAAAAGACGCGGAGGTACATTGTTAAGCAATGTAGGTCTGTTTAATCAGTTAGAGGAGCCCGATGCCAAATATTCTATATGCGATTTGGCATTTGGCCAATTTCAAGGTTCCTGGCACCAAGCATTTTCCTTGGGTGTTTGTTCGACTAATGTAAAGGGGATGAATATTGTTGTTGCTTCAACAAAGAATGTTGTTGGTAGTCAAGAATCTCTCGAAGAGCTTTGCTCCATTTACAAAGCTCTCCTTTTAGGCCCT**TCTAGA**GGGCCCTTCGAAGGTAAGCCTATCCCTAACCCTCTCCTCGGTCTCGATTCTACGCGTACCGGTCATCATCACCATCACCACCACCACCACCATTGA

>Atf2-V5-His_10_

**GGATCC**ATGGAAGATATAGAAGGATACGAACCACATATCACTCAAGAGTTGATAGACCGTGGCCATGCAAGACGTATGGGCCACTTGGAAAACTACTTTGCTGTTTTGAGTAGGCAGAAAATGTACTCGAATTTTACTGTTTACGCGGAATTGAATAAAGGTGTTAATAAGAGACAACTAATGCTTGTCTTGAAAGTATTACTTCAAAAATACTCAACTCTTGCGCATACAATCATTCCTAAGCATTATCCTCATCATGAAGCGTACTACTCTAGCGAAGAGTACCTTAGTAAACCTTTTCCACAGCATGATTTCATAAAGGTGATTTCTCATCTTGAATTCGATGACTTGATTATGAATAATCAACCAGAATACAGAGAAGTCATGGAGAAAATCTCAGAACAGTTCAAAAAGGATGATTTCAAAGTCACCAATAGGTTAATCGAATTGATTAGCCCTGTAATCATACCTCTGGGTAATCCGAAGAGGCCTAATTGGAGATTGATTTGTTTACCAGGTAAGGATACTGATGGGTTTGAAACGTGGAAAAACTTCGTTTATGTCACTAACCACTGCGGCTCCGACGGTGTCAGTGGATCGAATTTTTTCAAAGATTTAGCTCTACTCTTTTGTAAAATCGAAGAAAAAGGGTTTGATTATGATGAAGAGTTCATCGAAGATCAAGTCATCATTGACTATGATCGAGACTACACTGAAATTTCTAAATTGCCAAAACCGATTACGGATCGTATTGACTACAAGCCAGCATTGACTTCATTACCCAAATTCTTTTTAACAACCTTCATTTATGAACATTGTAATTTTAAAACCTCCAGCGAATCTACACTTACAGCTAGATATAGCCCCTCTAGTAATGCTAATGCTAGTTACAATTACTTGTTGCATTTCAGTACTAAGCAAGTAGAACAAATCAGAGCTCAGATCAAGAAAAATGTTCACGATGGGTGCACCCTAACACCCTTCATTCAAGCGTGCTTTCTTGTAGCCCTGTATAGACTGGATAAGCTGTTCACAAAATCTCTTCTCGAGTATGGGTTCGATGTGGCTATTCCAAGCAACGCAAGAAGGTTTTTACCAAACGATGAAGAGTTAAGAGATTCTTATAAATACGGCTCCAACGTTGGAGGTTCGCATTACGCCTATCTAATCTCCTCATTCGACATTCCCGAAGGTGACAATGACAAGTTTTGGAGTCTTGTCGAATACTACTATGACCGCTTTTTAGAATCGTACGACAACGGTGACCACTTGATTGGTCTGGGGGTCCTACAACTTGATTTTATCGTTGAAAACAAGAATATAGACAGCCTTCTTGCCAACTCTTATTTGCACCAGCAAAGAGGCGGTGCAATCATCAGTAATACAGGACTTGTCTCGCAAGATACGACCAAGCCGTACTACGTTCGGGATTTAATCTTCTCGCAGTCTGCAGGCGCCTTGAGATTTGCGTTCGGCCTAAACGTTTGCTCCACAAACGTGAATGGTATGAACATGGACATGAGCGTGGTTCAGGGCACTCTACGGGATCGTGGCGAATGGGAATCGTTCTGCAAGCTCTTCTACCAAACCATCGGCGAATTTGCGTCGCTT**TCTAGA**GGGCCCTTCGAAGGTAAGCCTATCCCTAACCCTCTCCTCGGTCTCGATTCTACGCGTACCGGTCATCATCACCATCACCACCACCACCACCATTGA

**Supplementary Figure 1**. Sequences of the recombinant constructs used in this work. Cloning into plasmid pYES2/CT introduces a V5 epitope and His_10_ tag at the C-terminus. The restriction enzyme sites used for cloning are underlined in bold.


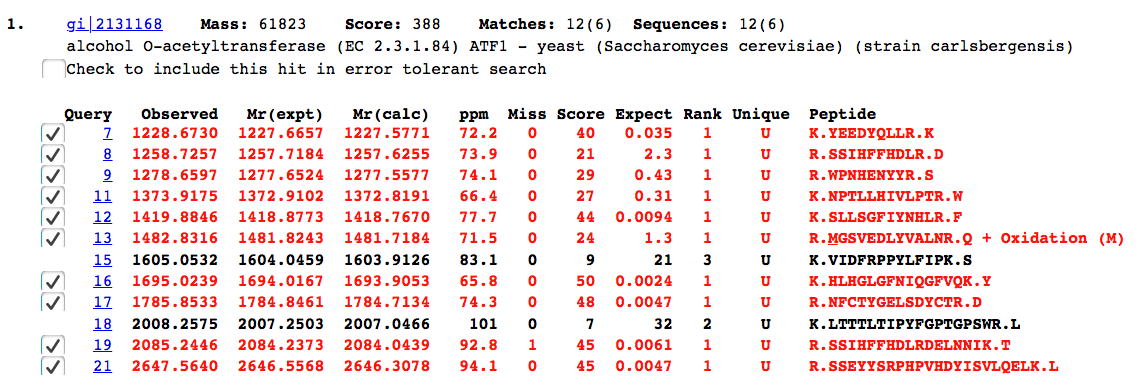


MNEIDEKNQA PVQQECLKEM IQNGHARRMG SVEDLYVALN RQNLYRNFCT YGELSDYCTR DQLTLALREI CLKNPTLLHI VLPTRWPNHE NYYRSSEYYS RPHPVHDYIS VLQELKLSGV VLNEQPEYSA VMKQILEEFK NSKGSYTAKI FKLTTTLTIP YFGPTGPSWR LICLPEEHTE KWKKFIFVSN HCMSDGRSSI HFFHDLRDEL NNIKTPPKKL DYIFKYEEDY QLLRKLPEPI EKVIDFRPPY LFIPKSLLSG FIYNHLRFSS KGVCMRMDDV EKTDDVVTEI INISPTEFQA IKANIKSNIQ GKCTITPFLH VCWFVSLHKW GKFFKPLNFE WLTDIFIPAD CRSQLPDDDE MRQMYRYGAN VGFIDFTPWI SEFDMNDNKE NFWPLIEHYH EVISEALRNK KHLHGLGFNI QGFVQKYVNI DKVMCDRAIG KRRGGTLLSN VGLFNQLEEP DAKYSICDLA FGQFQGSWHQ AFSLGVCSTN VKGMNIVVAS TKNVVGSQES LEELCSIYKA LLLGPSRGPF EGKPIPNP

LLGLDSTRTGHHHHHHHHHH

**Supplementary Figure 2.** Mass spectrometry of purified Atf1p excised from an SDS-PAGE gel. Top panel is a screenshot showing representative raw data from a MASCOT search of the NBCInr database for *S. cerevisiae* (126357 sequences). The lower panel shows the amino acid sequence of recombinant Atf1p with the sequence position of the 12 identified peptides highlighted in yellow.


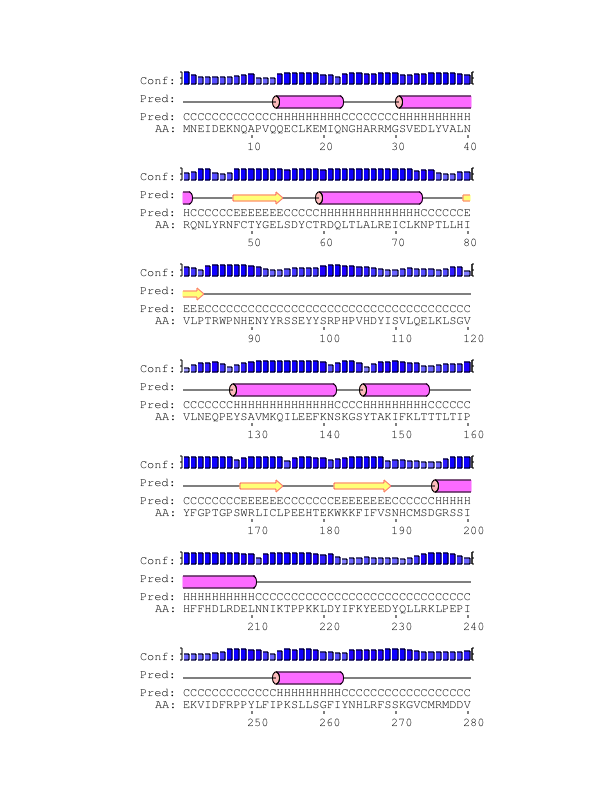

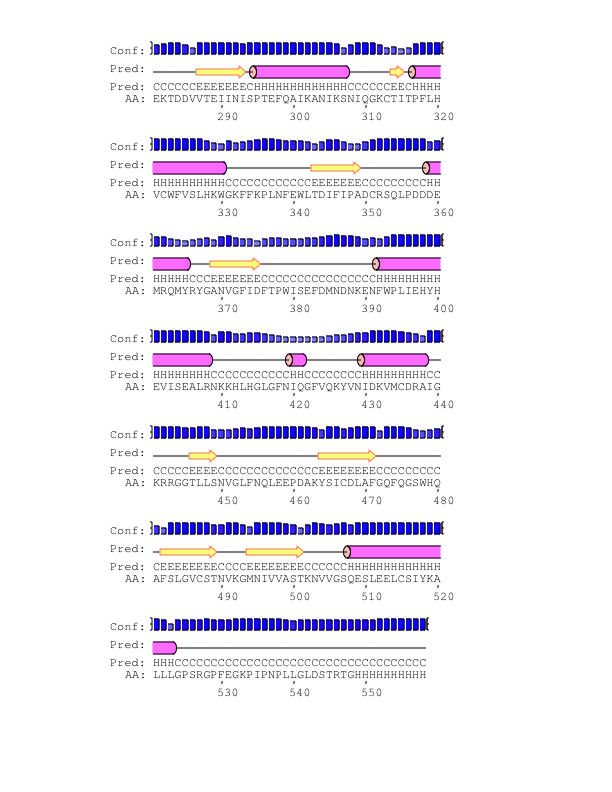

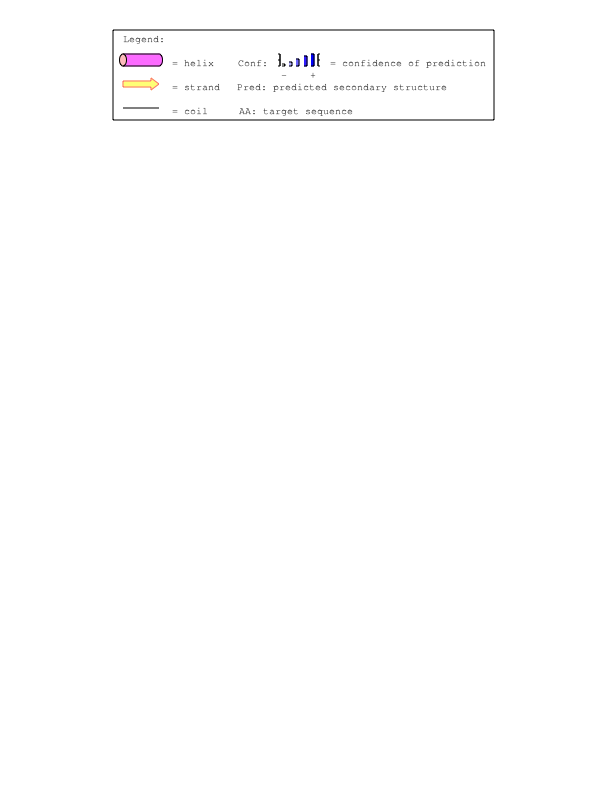


**Supplementary Figure 3.** Prediction of Atf1p secondary structure by the PSIPRED web server (http://bioinf.cs.ucl.ac.uk/psipred/)*^1, 2^***Supplementary Figure 4**


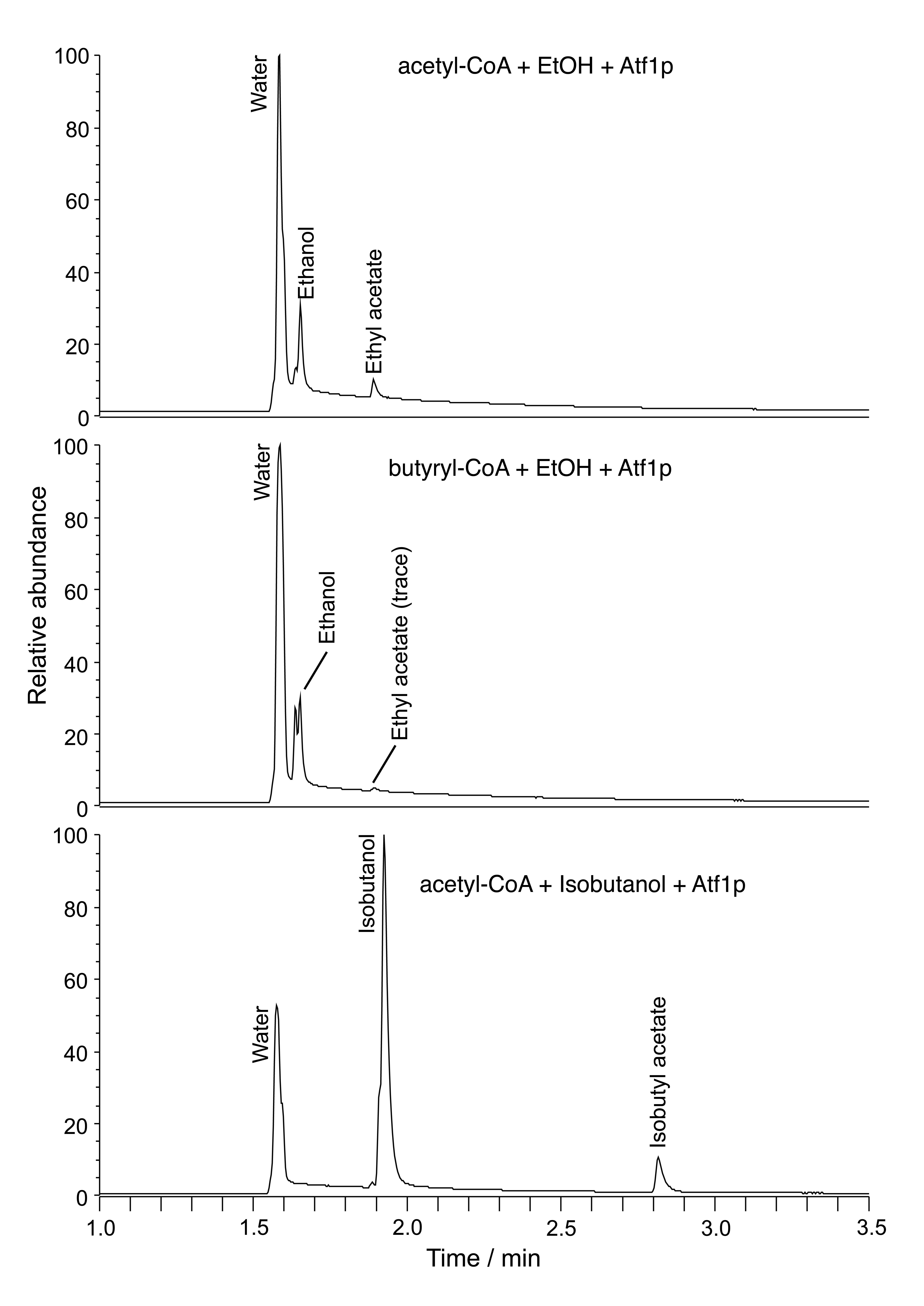


**Supplementary Figure 4**. GC-MS showing that Atf1p catalyses ethyl acetate and isobutyl acetate synthesis from acetyl-CoA and the corresponding alcohol, but does not synthesise esters from butyryl-CoA. These experiments were run at lower temperatures to identify low molecular weight volatile esters. *EtOH*, ethanol.

**Supplementary Figure 5**


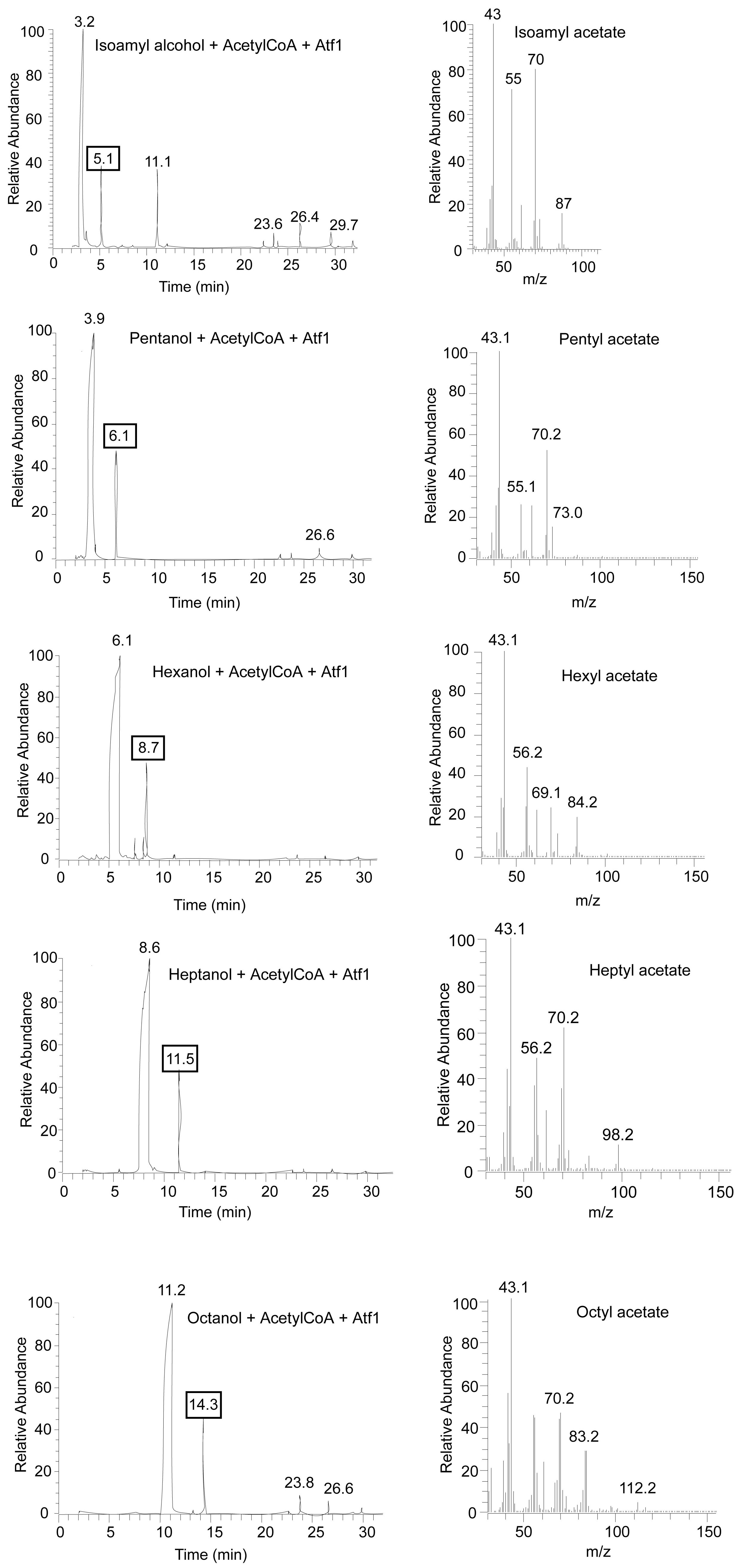


**Supplementary Figure 5**. GC-MS confirming that Atf1p is active in synthesizing a range of different acetate esters *in vitro*. *Left panels* are the GC traces for reactions as shown, *right panels* are the MS of the boxed peaks. Compounds are unambiguously identified from fragmentation patterns since the molecular ion is absent*^3^*. Characteristic peaks include the CH_3_CO rearrangement peak in all samples at *m/z =* 43, and the alkyl chain minus hydrogen (*m/z* of 70, 84, 98 and 112 for linear pentyl, hexyl, heptyl and octyl, respectively). See legend of Figure 3 for further information.

**Supplementary Figure 6**


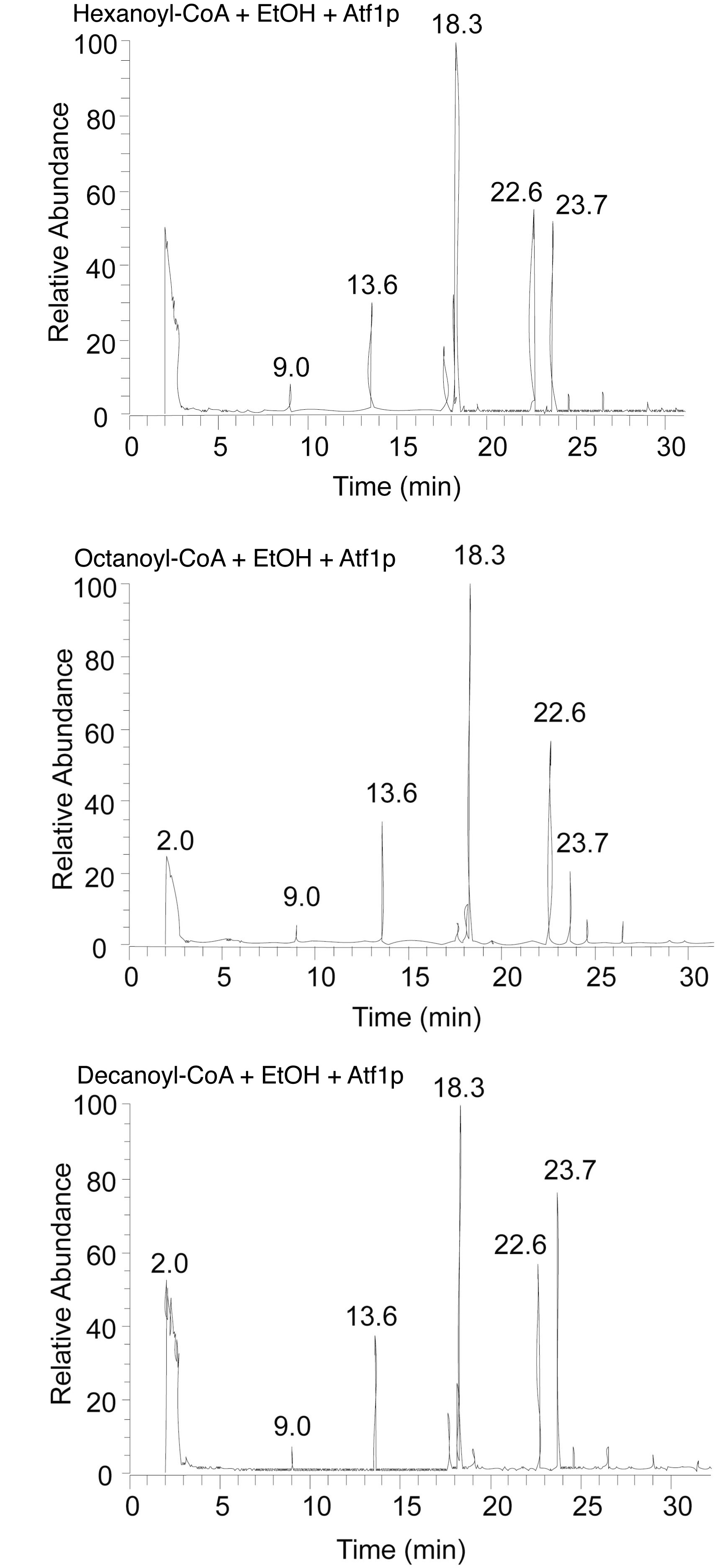


**Supplementary Figure 6**. GC-MS shows that Atf1p is not active in synthesizing esters from other acyl-CoAs. Only background peaks are observed in all of the examples shown. Although neither of the reactants can be observed, the same method was previously able to detect medium-chain fatty acid ethyl ester products synthesized by the acyltransferase Eht1p*^4^*. The hypothetical products ethyl hexanoate, ethyl octanoate and ethyl decanoate would be expected to elute at 6.4 min, 11.7 min and 16.9 min, respectively. *EtOH*, ethanol. Note that the six background peaks consistently observed at 2.0, 9.0, 13.6, 18.3, 22.6 and 23.7 min are artificially scaled and are actually at very low absolute abundance.


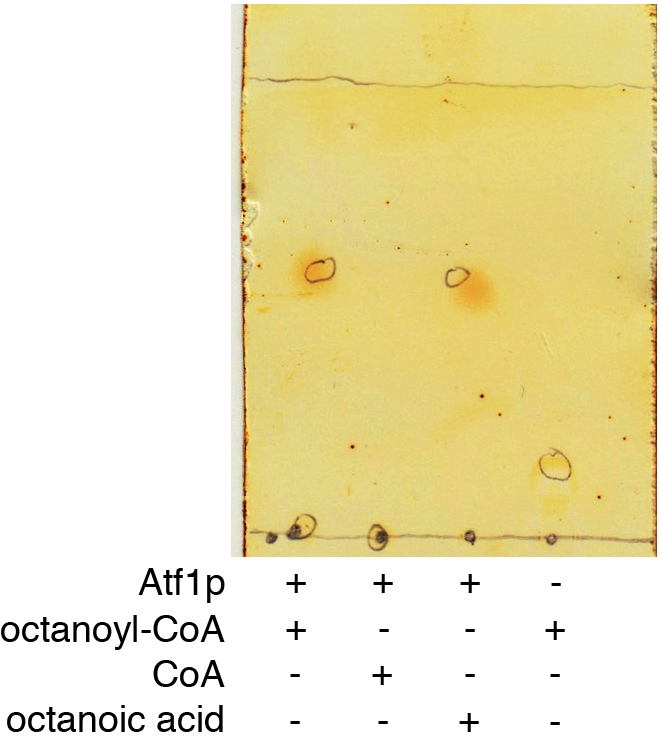


**Supplementary Figure 7.** Thin-layer chromatography confirms that Atf1p hydrolyses octanoyl-CoA to CoA and octanoic acid. The reaction between Atf1p and octanoyl-CoA is compared to control experiments using CoA (which does not migrate under the solvent conditions used) and octanoic acid standards. A further control demonstrates that octanoyl-CoA is stable in the absence of enzyme. All reactants were at 2.5 mM with enzyme at 3.5 μM where present.

**Supplementary Table 1**. Kinetic data from coupled enzyme assays. Errors are from curve-fitting of intitial (linear) rates to the Michaelis-Menten equation.

|  | **Atf1p wild-type** | | | **Atf1p H191A** | | |
| --- | --- | --- | --- | --- | --- | --- |
| **Acyl-CoA** | ***k_cat_* (s^-1^)** | ***K_M_* (μM)** | ***k_cat_*/*K_M_* (M^-1^s^-1^)** | ***k_cat_* (s^-1^)** | ***K_M_* (μM)** | ***k_cat_*/*K_M_* (M^-1^s^-1^)** |
| Acetyl | 0.4 ± 0.02 | 61 ± 9 | 6656  ± 1000 | 0.2 ± 0.01 | 40 ± 3 | 4859  ± 339 |
| Butyryl | 1 ± 0.01 | 25 ± 1 | 41468  ± 1050 | 0.4 ± 0.01 | 42 ± 2 | 9832  ± 535 |
| Hexanoyl | 0.4 ± 0.03 | 12 ± 3 | 29106  ± 8033 | 0.2 ± 0.01 | 12 ± 1 | 18803  ± 948 |
| Octanoyl | 0.3 ± 0.01 | 3 ± 1 | 75758  ± 11872 | 0.2 ± 0.01 | 5 ± 1 | 35673  ± 3479 |
| Decanoyl | 1 ± 0.1 | 23 ± 7 | 43421  ± 14896 | 0.3 ± 0.01 | 14 ± 1 | 17847  ± 1622 |
| Dodecanoyl | 0.2 ± 0.03 | 6 ± 2 | 40000  ± 15868 | 0.1 ± 0.01 | 108 ± 18 | 1291  ± 252 |

**Supplementary References.**

[1] Buchan, D., Minneci, F., Nugent, T., Bryson, K., and Jones, D. T. (2013) Scalable web services for the PSIPRED protein analysis workbench, *Nucleic Acids Res* *41*, W430-438.

[2] Jones, D. T. (1999) Protein secondary structure prediction based on position-specific scoring matrices, *J Mol Biol* *292*, 195-202.

[3] Sharkey, A. G., Shultz, J. L., and Friedel, R. A. (1959) Mass spectra of esters: formation of rearrangement ions, *Anal Chem* *31*, 87-94.

[4] Knight, M. J., Bull, I. D., and Curnow, P. (2014) The yeast enzyme Eht1 is an octanoyl-CoA:ethanol acyltransferase that also functions as a thioesterase, *Yeast* *31*, 463-474.
